# Are psychological symptoms a risk factor for musculoskeletal pain in adolescents?

**DOI:** 10.1007/s00431-021-04002-5

**Published:** 2021-03-02

**Authors:** Alessandro Andreucci, Paul Campbell, Kate M. Dunn

**Affiliations:** 1 Primary Care Centre Versus Arthritis, School of Primary, Community and Social Care, Keele University, Staffordshire, ST5 5BG UK; 2 Center for General Practice at Aalborg University, Department of Clinical Medicine, Aalborg University, 9220 Aalborg, Denmark; 3 Midlands Partnership NHS Foundation Trust, Department of Research and Innovation, St Georges Hospital, Corporation Street, Stafford, Staffordshire ST16 3SR UK

**Keywords:** Internalizing, Externalizing, ALSPAC, Prospective study, Musculoskeletal pain, Adolescent

## Abstract

Adolescent musculoskeletal pain is common and is associated with musculoskeletal pain in adulthood. Psychological symptoms, also common in adolescence, have been shown to be associated with musculoskeletal pain, but the current evidence is mixed and may be dependent on effect modifiers. This study investigated whether adolescents with psychological symptoms (internalizing and externalizing constructs) at age 13 years were at higher odds for musculoskeletal pain at age 17 years and whether the associations were modified by pubertal status and sex. A prospective cohort design examined data on 3865 adolescents from the Avon Longitudinal Study of Parents and Children (ALSPAC). Associations between baseline (aged 13 years) internalizing and externalizing symptoms and musculoskeletal pain at follow-up (aged 17 years) were investigated using logistic regression producing odds ratios (OR) and 95% confidence intervals (95% CI). In total 43.1% of adolescents reported musculoskeletal pain at follow-up. Externalizing symptoms at baseline increased the odds of musculoskeletal pain (OR 1.68, 95% CI 1.28, 2.20), and internalizing symptoms demonstrated a non-significant increase (OR 1.26, 95% CI 0.98, 1.62). Effect modification analysis showed an increased effect dependent on pubertal status.

*Conclusion*: Adolescents with externalizing symptoms, and to some extent internalizing symptoms, are at increased odds of later musculoskeletal pain. Future research is now required to understand the reasons for these associations.

**Table:** 

**What is Known:** • *Current evidence regarding the association between internalizing symptoms and externalizing symptoms and future musculoskeletal pain in adolescents is mixed.*	
**What is New:** • *This study found that adolescents with externalizing symptoms, and to some extent internalizing symptoms, are at increased odds for musculoskeletal pain, with an increased influence dependent on pubertal status.* • *These results are of interest for the development of timely preventative interventions designed to reduce the risk of musculoskeletal pain.*

**Supplementary Information:**

The online version contains supplementary material available at 10.1007/s00431-021-04002-5.

## Introduction

Musculoskeletal pain is common in adolescence, with estimates up to 40% globally [1]. Adolescent musculoskeletal pain is associated with a high burden in terms of years lived with disability [2] and later musculoskeletal pain in adulthood. Evidence suggests adolescence is a potential sensitive life period for the development of future musculoskeletal pain, and greater understanding is required of potential risk factors at this age [3]. One group of risk factors investigated are psychological symptoms, which are also common in adolescence [4, 5]. Psychological symptoms might exert their effect on musculoskeletal pain through several mechanisms, including a dysfunction of the HPA axis [6–9], an effect on brain regions (amygdala, anterior insula) involved the emotional-affective processing of pain which might result in a decreased pain threshold [10, 11] and increased levels of pro-inflammatory cytokines (IL-6, IL-1β and TNF-α) involved in pain processing [11–13]. Previous studies demonstrated associations between psychological symptoms and musculoskeletal pain, but findings are inconsistent [14–20]. There are several potential explanations for this inconsistency. One is psychological symptom type, with differences reported dependent on whether symptoms are internalizing (e.g. depression and anxiety) or externalizing (e.g. attention-deficit-hyperactivity disorders and behavioural problems) [14–16, 18, 19]. Another explanation is potential effect modification dependent on sex; one of three studies [17, 19, 20] investigating sex reported a significant association for internalizing symptoms in girls but not boys, and for externalizing symptoms in boys but not girls [20]. In addition, internalizing symptoms are more common in girls, whereas externalizing symptoms are more common in boys [4, 21]. A further potential effect modifier is pubertal status; research shows that individuals with advanced pubertal development may be at higher risk for musculoskeletal pain onset [22], and experiencing puberty at a different pace (early or late development) compared to the peer average may increase the risk of developing psychological symptoms [23]. The aim of this study was therefore to prospectively test whether internalizing and externalizing symptoms in adolescents were predictive of musculoskeletal pain and to investigate whether sex and pubertal status modify these associations.

## Materials and methods

### Design

The study was a secondary data analysis of a longitudinal prospective cohort study.

### Participants

**Fig. 1:**
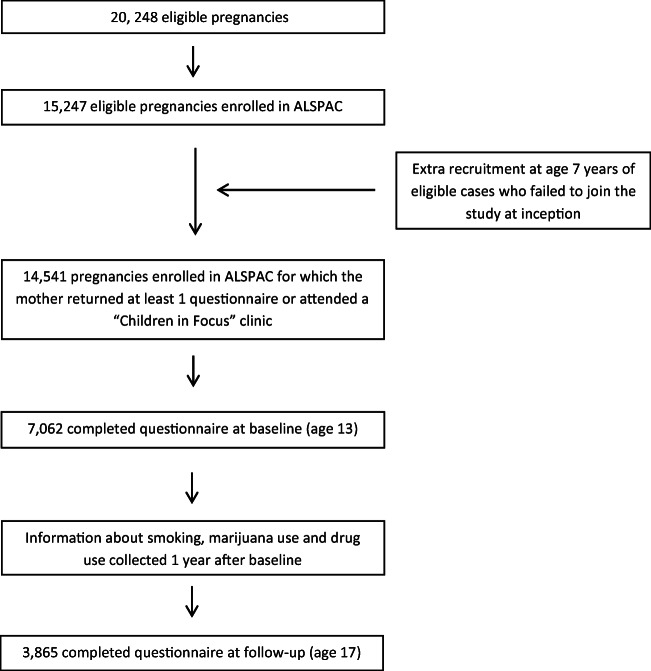
Flowchart describing the number of adolescents included in this current study

The study population was recruited to the Avon Longitudinal Study of Parents and Children (ALSPAC), a birth cohort study. All pregnant women resident in Avon (South West England) who were expected to give birth between 1 Apr. 1991 and 31 Dec. 1992 were eligible to enrol in the study [24]. Information on parents and children was collected during pregnancy and throughout childhood using postal questionnaires and clinical visits [24]. The initial number of pregnant women enrolled, and for which the mother returned at least 1 questionnaire or attended a “Children in Focus” clinic, was 14,541 (see Fig. 1). In the current analysis, adolescents were 13 years old at baseline and 17 years old at follow-up. This selection was made based on the availability of variables. Further information about the study and data collection is described in the study protocol [24]. The ALSPAC study website contains details of all the data (http://www.bristol.ac.uk/alspac/researchers/our-data/).

### Measures

#### Exposures

Internalizing and externalizing symptoms were assessed through parent report at baseline using the Strengths and Difficulties Questionnaire (SDQ). The SDQ is a 25-item questionnaire with five subscales: emotional problems, peer problems, behavioural problems, hyperactivity and prosocial behaviour [25]. Each subscale includes 5 questions rated on a 3-point scale (“Not true” = 0, “Somewhat true” = 1, “Certainly true” = 2) producing a score range from 0 to 10. The emotional problems and peer problems subscales were combined to create the internalizing construct (range 0 to 20), and the behavioural problems and hyperactivity subscales were combined to create the externalizing construct (range 0 to 20). This approach of combining the subscales into broader “internalizing” and “externalizing” constructs has been shown to be suitable and valid for use in epidemiological studies within adolescent cohorts at low risk of psychological symptoms [26, 27]. A 10% clinical cut-off was used for defining adolescents with “abnormal” levels of internalizing and externalizing symptoms, in order to identify cases of clinical relevance and reduce the rate of false-positive cases in a low-risk sample, following previous methodology [25, 28]. The SDQ has shown satisfactory reliability (Cronbach *α*, 0.82; retest stability after 4 to 6 months, 0.72 for the total difficulties scale) [29] and similar performance compared to the Child Behavioural Checklist for the identification of internalizing and externalizing symptoms [30].

#### Outcome

Pain presence at follow-up was assessed through the question “have you had any aches or pains that have lasted for a day or longer in the past month?” This question was supported by a manikin (pictorial description of body areas that includes two diagrams, one for the front and one for the back) as well as the sentence “Please shade in the diagrams to show where exactly you felt the pain(s)”. Pain manikins have previously been shown to be valid and reliable within population cohorts [31–33]. The pain question and manikin response were used to create an outcome variable that represented the presence of musculoskeletal pain at follow-up (pain that related only to the head or abdomen was excluded), following methodology of a previous study carried out with the ALSPAC cohort [34]. Participants were classified as “having musculoskeletal pain” or “not having musculoskeletal pain”.

#### Potential effect modifiers

Sex and pubertal stage were included as potential effect modifiers. Pubertal stages were measured at baseline using 5-point rating scales and categorized in Tanner stages (from 1 to 5) according to the parental responses. The questionnaire included two scales, and parents indicated the stage (1 to 5) of development their child had reached in each scale. The highest of two ratings (breast development or pubic hair for girls; genital development or pubic hair for boys) was used to indicate the pubertal stage. Adolescents were grouped in the early/beginning puberty group (Tanner stage = 1 or 2), mid/advanced puberty group (Tanner stage = 3 or 4) and post-pubertal group (Tanner stage = 5).

#### Potential confounders

Potential confounders identified from previous literature were physical activity, smoking, marijuana use and drug use [18, 35, 36]. Parent-reported information on physical activity was gathered at baseline through a questionnaire that included five options of vigorous physical activity (e.g. running, football, swimming, athletics) frequency “none/less than once a week/1–3 times a week/4–6 times a week/daily”. Adolescents who performed physical activity “4–6 times a week” or “daily” were considered as having “high levels of physical activity”, and those who performed physical activity “none”, “less than once a week” or “1–3 times a week” as having “low levels of physical activity”. Smoking (yes/no), marijuana use (yes/no) and drug use (yes/no) were self-reported by adolescents at age 14.

### Data analysis

Baseline descriptive analysis was performed and values were shown as means and standard deviations (SD) or counts (%) where appropriate. The association between internalizing and externalizing symptoms (defined as internalizing and externalizing scores ≥ 90th percentile) at baseline and musculoskeletal pain at follow-up was assessed by logistic regression producing odds ratios (OR) with 95% confidence intervals (CI). Associations were adjusted for potential confounders. Potential effect modifiers were examined by statistical interaction test and analysis stratified by sex and pubertal stage. Comparisons were made between stratified groups to observe actual differences in the magnitude or direction of the association between psychological symptoms and musculoskeletal pain across strata of the effect modifier. Potential bias due to missing data was assessed by inspecting the percentage of missingness for each variable, and missing data were replaced through a chained equation multiple imputation method in order to maximize statistical power and increase precision (i.e. to limit the possibility of a biased estimate) [37, 38]. All raw variables used in the analysis (i.e. internalizing, externalizing, sex, puberty, physical activity, smoking, marijuana, drug use) were included in the imputation model [37, 38]. The outcome was included in the imputation model, but not imputed. A number of datasets (*n* = 35) higher than the highest percentage of missing data among the variables (33% for the variable “puberty”) were created [37, 38]. All data was checked for distribution and multicollinearity prior to data analysis*.* All statistical analyses were carried out using STATA 14.

## Results

### Recruitment

A total of 7062 adolescents were present at baseline, of which 3522 (49.9%) were boys and 3540 (50.1%) girls. At follow-up, 3865 adolescents responded, representing 54.7% response.

### Participant characteristics

Table 1 outlines the baseline characteristics of the cohort. The mean and median internalizing scores were 2.6 (± 2.8) and 2 (interquartile range, IQR, 0–17), respectively. Boys and girls had similar mean (boys 2.6 ± 2.8, girls 2.7 ± 2.7) and median (boys (2, IQR, 0–17), girls (2, IQR, 0–15)) internalizing scores and similar proportions above the 90th percentile cut-off (boys 9.7% vs. girls 9.8%). The mean and median externalizing scores were 4.2 (± 3.2) and 4 (IQR, 0–18), respectively. Boys had higher mean (4.6 ± 3.3) and median (4, IQR, 0–18) externalizing scores compared to girls (mean 3.7 ± 2.9, median 3, IQR, 0–17) and were more likely to be defined as having externalizing symptoms (boys 12.7% vs. girls 7.2%). Girls were in a more advanced pubertal status compared to boys (25.9% vs. 11.0% in the post-pubertal stage, respectively). Approximately 26% of adolescents reported having ever tried smoking (18.9% of boys and 31.3% of girls), whilst 8.6% reported having ever tried marijuana (similar between boys and girls). Approximately 15% of adolescents tried any type of drugs ever, with more girls (17.7%) than boys (12.5%). Finally, approximately 45% of adolescents performed physical activity more than 3 times a week, with more boys (53.9%) than girls (37.6%). At follow-up, 1666 adolescents (43.1%) reported the presence of musculoskeletal pain, whilst 2199 (56.9%) did not report musculoskeletal pain.

**Table 1:** Baseline sample characteristics

Psychological characteristics	Boys	Girls	Overall	Missing
Internalizing score (mean ± SD)	2.6 (± 2.8)	2.7 (± 2.7)	2.6 (± 2.8)	20.5%
Internalizing score (median, IQR)	2 (0–17)	2 (0–15)	2 (0–17)	
Externalizing score (mean ± SD)	4.6 (± 3.3)	3.7 (± 2.9)	4.2 (± 3.2)	20.5%
Externalizing score (median, IQR)	4 (0–18)	3 (0–17)	4 (0–18)	
Psychological symptoms > 90th percentile	Boys	Girls	Overall	Missing
Internalizing	343 (9.7%)	346 (9.8%)	689 (9.8%)	20.5%
Externalizing	446 (12.7%)	256 (7.2%)	702 (9.9%)	20.5%
Effect modifiers	Boys	Girls	Overall	Missing
Sex	3168 (45.4%)	3803 (54.6%)	6971	0%
Pubertal stage	Boys	Girls	Overall	Missing
Pre-early puberty	389 (15.8%)	240 (7.8%)	629 (11.4%)	33.2%
Mid/advanced puberty	1803 (73.2%)	2027 (66.3%)	3830 (69.4%)	33.2%
Post-puberty	272 (11.0%)	791 (25.9%)	1063 (19.2%)	33.2%
Confounders	Boys	Girls	Overall	Missing
Cigarette smoking (yes)	489 (18.9%)	1038 (31.3%)	1527 (25.9%)	26.2%
Marijuana smoking (yes)	216 (8.3%)	294 (8.8%)	510 (8.6%)	25.9%
Drug use ever (yes)	328 (12.5%)	592 (17.7%)	920 (15.4%)	25.4%
Physical activity (*> 3 times a week)*	1490 (53.9%)	1175 (37.6%)	2665 (45.2%)	30.5%

*SD* standard deviation, *IQR* interquartile range

### Characteristics of adolescents lost to follow-up

A total of 3197 adolescents (45.3%) were lost to follow-up. Those lost were significantly more likely to be boys (54.9% vs. 41.9%) and smokers (27.2% vs. 22.3%) and had higher internalizing (10.4% vs. 8.9%) and externalizing symptoms (12.0% vs. 7.3%) compared to those completing follow-up.

### Association between internalizing symptoms at baseline and musculoskeletal pain at follow-up

Adolescents with internalizing symptoms at baseline were at increased odds of musculoskeletal pain at follow-up, although this association was not significant (adjusted OR 1.26; 95% CI 0.98, 1.62); see Table 2. Stratification by sex showed similar estimates of risk between boys and girls (boys (adj. OR 1.18; 95% CI 0.81, 1.71), girls (adj. OR 1.34; 95% CI 0.96, 1.88)); the interaction test was not significant (Table 2). Analysis stratified by pubertal stages showed that the association between internalizing symptoms and musculoskeletal pain was stronger in early pubertal stages than later stages, although this was statistically non-significant (early pubertal stage (adj. OR = 2.27; 95% CI 0.99, 5.20), mid/advanced pubertal stage (adj. OR = 1.17; 95% CI 0.87, 1.57), post-pubertal stage (adj. OR = 1.21; 95% CI 0.70, 2.10)). The interaction test for puberty was not significant (Table 2).

**Table 2:** Logistic regression of the association between internalizing at baseline and musculoskeletal pain at follow-up

Unadjusted analysis		
Musculoskeletal pain at follow-up	Odds ratio	95% CI
Overall (*N* = 3865)	1.17	0.92, 1.50
Adjusted analysis*		
Musculoskeletal pain at follow-up	Odds ratio	95% CI
Overall (*N* = 3865)	1.26	0.98, 1.62
Analysis stratified by sex		
Unadjusted analysis		
Musculoskeletal pain at follow-up	Odds ratio	95% CI
Girls (*N* = 2245)	1.26	0.90, 1.75
Boys (*N* = 1620)	1.09	0.76, 1.56
Adjusted analysis*		
Musculoskeletal pain at follow-up	Odds ratio	95% CI
Girls (*N* = 2245)	1.34	0.96, 1.88
Boys (*N* = 1620)	1.18	0.81, 1.71
Interaction term*		
Musculoskeletal pain at follow-up	Odds ratio	95% CI
Girls # internalizing	1.16	0.71, 1.89
Analysis stratified by pubertal stages		
Unadjusted analysis		
Musculoskeletal pain at follow-up	Odds ratio	95% CI
Early pubertal stage (*N* = 378)^●^	2.13	0.97, 4.67
Mid/advanced pubertal stage (*N* =2663)^●●^	1.09	0.82, 1.46
Post-pubertal stage (*N* = 725)^●●●^	1.09	0.63, 1.86
Adjusted analysis*		
Musculoskeletal pain at follow-up	Odds ratio	95% CI
Early pubertal stage (*N* = 378)^●^	2.27	0.99, 5.20
Mid/advanced pubertal stage (*N* =2663)^●●^	1.17	0.87, 1.57
Post-pubertal stage (*N* = 725)^●●●^	1.21	0.70, 2.10
Interaction term*		
Musculoskeletal pain at follow-up	Odds ratio	95% CI
Mid/advanced puberty # internalizing	0.50	0.22, 1.17
Post-puberty # internalizing Reference group: early puberty	0.51	0.20, 1.30

^**●**^Sample size varies between 378 and 446 as a result of multiple imputation

^**●●**^Sample size varies between 2663 and 2729 as a result of multiple imputation

^**●●●**^Sample size varies between 725 and 784 as a result of multiple imputation

*Analysis adjusted for smoking, marijuana use, drug use and physical activity

### Association between externalizing symptoms at baseline and musculoskeletal pain at follow-up

Adolescents with externalizing symptoms at baseline were at significantly higher odds of musculoskeletal pain at follow-up (adj. OR = 1.68; 95% CI 1.28, 2.20); see Table 3. Analysis stratified by sex showed similar estimates of risk in boys and girls (boys (adj. OR 1.88; 95% CI 1.29, 2.75), girls (adj. OR 1.61; 95% CI 1.09, 2.40)), and the interaction test was not significant (Table 3). Further stratified analysis showed that the association between externalizing symptoms and musculoskeletal pain was stronger among adolescents in mid/advanced pubertal stage compared to those at the early or post-pubertal stage (early pubertal stage (adj. OR = 1.20; 95% CI 0.48, 3.03), mid/advanced pubertal stage (adj. OR = 1.85; 95% CI 1.33, 2.57), post-pubertal stage (adj. OR = 1.45; 95% CI 0.76, 2.78)) (Table 3). The interaction test for puberty was not significant (Table 3).

**Table 3:** Logistic regression of the association between externalizing at baseline and musculoskeletal pain at follow-up

Unadjusted analysis		
Musculoskeletal pain at follow-up	Odds ratio	95% CI
Overall (*N* = 3865)	1.78	1.37, 2.32
Adjusted analysis*		
Musculoskeletal pain at follow-up	Odds ratio	95% CI
Overall (*N* = 3865)	1.68	1.28, 2.20
Analysis stratified by sex		
Unadjusted analysis		
Musculoskeletal pain at follow-up	Odds ratio	95% CI
Girls (*N* = 2245)	1.70	1.14, 2.51
Boys (*N* = 1620)	1.96	1.35, 2.84
Adjusted analysis*		
Musculoskeletal pain at follow-up	Odds ratio	95% CI
Girls (*N* = 2245)	1.61	1.09, 2.40
Boys (*N* = 1620)	1.88	1.29, 2.75
Interaction term*		
Musculoskeletal pain at follow-up	Odds ratio	95% CI
Girls # externalizing	0.85	0.49, 1.47
Analysis stratified by pubertal stages		
Unadjusted analysis		
Musculoskeletal pain at follow-up	Odds ratio	95% CI
Early pubertal stage (*N* = 378)^●^	1.33	0.55, 3.20
Mid/advanced pubertal stage (*N* =2663)^●●^	1.93	1.39, 2.68
Post-pubertal stage (*N* = 725)^●●●^	1.54	0.81, 2.94
Adjusted analysis*		
Musculoskeletal pain at follow-up	Odds ratio	95% CI
Early pubertal stage (*N* = 378)^●^	1.20	0.48, 3.03
Mid/advanced pubertal stage (*N* =2663)^●●^	1.85	1.33, 2.57
Post-pubertal stage (*N* = 725)^●●●^	1.45	0.76, 2.78
Interaction term*		
Musculoskeletal pain at follow-up	Odds ratio	95% CI
Mid/advanced puberty # externalizing	1.50	0.58, 3.89
Post-puberty # externalizing Reference group: early puberty	1.20	0.39, 3.68

^**●**^Sample size varies between 378 and 446 as a result of multiple imputation

^**●●**^Sample size varies between 2663 and 2729 as a result of multiple imputation

^**●●●**^Sample size varies between 725 and 784 as a result of multiple imputation

*Analysis adjusted for smoking, marijuana use, drug use and physical activity

## Discussion

### Main findings

Psychological symptoms were associated with increased odds of reporting musculoskeletal pain, with a 26% non-significant increase in odds for internalizing symptoms and a statistically significant 68% increase for externalizing symptoms. Effect modification analysis showed no effect for sex, but stratification by pubertal status showed some non-significant effect modification trends with increased effect of internalizing in early pubertal stages and increased effect of externalizing in mid/advanced pubertal stage.

### Comparison with previous literature

Musculoskeletal pain was common in this cohort (> 40%), in agreement with findings of other cohorts that used similar pain assessment methods [39]. Whilst previous research has shown mixed effects of psychological symptoms on later adolescent musculoskeletal pain [14–20], there is support for the current findings. Two studies [14, 15] also using the SDQ to evaluate psychological symptoms report a similar significant effect for musculoskeletal pain among those with behavioural problems (part of the externalizing construct), and other recent studies have shown analogous effects using similar birth cohort data [40] and primary care consultation data [41]. However, like-for-like comparison with previous research in adolescent populations can be problematic due to differences in the ages studied, as adolescence is a period of substantial physical and cognitive change [4, 5, 23], plus differences in psychological symptom measurement [18–20], pain measurement (e.g. different pain sites, acute or chronic pain) [16, 17, 20] and differences in time intervals between exposure and outcomes [16, 17, 20].

No sex differences were shown in the association between musculoskeletal pain and either internalizing or externalizing symptoms, in agreement with two [17, 19] out of three previous studies where analyses were stratified by sex, suggesting it is not a significant effect modifier. Regarding pubertal status, the time of onset of puberty might increase the risk of psychological symptom development during adolescence [23] and therefore increase the risk of musculoskeletal pain. Results from this current study partly agree with this hypothesis, as different estimates of risk were observed across different pubertal stages (although interaction terms were not significant). However, further research is required to investigate mechanisms underlying different directions of effect for internalizing and externalizing symptoms.

### Strengths and limitations

A major strength of this study is the prospective cohort design in a large representative population sample of adolescents. This allowed interpretation of findings that can take account of the temporal sequence between exposure and outcome [42], enabled testing of interaction and facilitated adjustment for confounders. Another strength is the examination of potential effect modifiers, which firstly help to untangle the current mixed evidence within the literature and secondly identify adolescent groups at greater or lesser levels of risk. A further strength is the use of the SDQ, which is a valid and suitable measure for the parent report of the adolescents’ behavioural and emotional disorders [25, 27, 43, 44]. Some limitations are also present. The musculoskeletal pain measure did not include assessments of pain intensity or function, which may have given a clearer indication of impact [45]. Furthermore, the same measure for the assessment of musculoskeletal pain was not available at baseline, and this is a major limitation. However, sensitivity analysis with adjustment for an alternative measure of pain collected at baseline (only pain in the arms and/or legs was assessed) showed similar results (available in supplementary file), suggesting that previous pain presence is unlikely to explain the reported effects, notably that externalizing symptoms are associated with later musculoskeletal pain presence. There are limitations with regard to the assessment of puberty. The gold standard is a physical examination, and although parental report is acceptable, some misclassification may be present specifically regarding the accuracy in the pubertal assessment of boys compared to girls [46]. This was a prospective study with a 4-year follow-up, and it was not possible to assess whether the psychological status of adolescents changed between baseline and follow-up (with potential differences among boys and girls) [23]. However, previous studies have used similar approaches with a 4-year follow-up [34, 47], and the gap between measurements is still relevant when taking a long-term or life-course approach to studying pain, which is recommended for musculoskeletal pain [3, 48]. In addition, it might be argued that the presence of chronic diseases and treatments (e.g. complex regional pain syndromes, chronic fatigue syndrome, JIA, diabetes, pharmacological treatments) may have influenced the results found, as they can be associated both with musculoskeletal pain and psychological symptoms [49]. However, given the generally low prevalence of these conditions and treatments at a population level [50–56], this is unlikely to affect the results found. Other additional uncontrolled factors might have affected the results found, such as genetic factors, familiar problems (including socio-economic or health issues) [20, 57–61] and sleep problems, which are associated with musculoskeletal pain in children and adolescents [39, 41]. In addition, whilst we controlled for physical activity at baseline, we cannot exclude that pain was the result of overuse injury or of intensive physical activity sessions in adolescents which might result in transient pain [62–64]. Furthermore, although adjustment for baseline physical activity was applied within the analysis, we were unable to account for any potential changes in physical activity from baseline to follow-up, which has been documented within previous research [65]. Finally, more than 45% of adolescents were lost to follow-up, and those lost to follow-up were significantly more likely to be boys and smokers and had higher internalizing and externalizing symptoms compared to completers. This may have affected the estimates of association (i.e. towards an underestimation of effect), if those lost to follow-up were at increased odds for musculoskeletal pain compared to those who completed.

### Interpretation and implications

The findings of this study might be interpreted via the biopsychosocial model of pain. From a biological perspective, the stress originating from the presence of psychological symptoms might result in overstimulation of the HPA system and dysfunctional cortisol production [8, 66, 67], which has been shown to be associated with pain and increased perception of pain [8, 67]. Psychological symptoms also affect brain regions (amygdala, anterior insula) involved in the emotional-affective processing and interpretation of pain, which can decrease the pain threshold [10, 11, 68]. In addition, increased levels of pro-inflammatory cytokines (IL-6, IL-1β and TNF-α), which contribute to neuro-immune interactions involved in the pain processing [11–13], have been observed after psychological stress in laboratory studies [69] and 2 years after the assessment of internalizing and externalizing symptoms in a study using the ALSPAC cohort [70]. These mechanisms might operate alone or in combination and might be further enhanced by behavioural factors such as rumination and attention, early life adversities (e.g. experiences of physical, emotional and sexual abuse) [9, 71] and social factors (low socio-economic status, parental health conditions), which might foster the development of externalizing symptoms that precede musculoskeletal pain [20, 57, 59, 72–75]. At a psychological level, mood symptoms and stress can lead to maladaptive thought processing (catastrophizing and fear avoidance) leading to greater sensitivity to the perception of pain [76, 77]. Adolescents with externalizing symptoms might also be more physically active and engage in high-risk behaviours (e.g. alcohol consumption, physical conflict with peers) [36], which may increase exposure to activities associated with injury and resulting pain. This study has given greater understanding of the relationship between psychological symptoms and pain development in adolescence and highlighted potential modification effects of puberty. More longitudinal research that can track adolescents over time at multiple time points is now required to understand the causal pathways in order to develop appropriate and timely interventions to reduce the risk of musculoskeletal pain and its recurrence/persistence in adolescents.

## Conclusions

This study found that adolescents with externalizing symptoms are at increased risk of musculoskeletal pain 4 years later. Future research is required to understand the mechanisms that underpin this association, to lay the basis for potential intervention development in this population.

## Supplementary Information

ESM 1 (DOCX 14 kb)

## Abbreviations

ALSPAC: Avon Longitudinal Study of Parents and Children
SDQ: Strengths and Difficulties Questionnaire
OR: Odds ratio
95% CI: 95% confidence intervals
