# Supplementary material for: Are psychological symptoms a risk factor for musculoskeletal pain in adolescents?
Source: Eur J Pediatr. 2021 Mar 2;180(7):2173–83. doi: 10.1007/s00431-021-04002-5 (PMC8195761; doi:10.1007/s00431-021-04002-5)
Supplement: Supplementary file 1 — (DOCX 14 kb) [file 431_2021_4002_MOESM1_ESM.docx]

**Supplementary file**

**Results of the analysis of the association between internalizing symptoms at baseline and musculoskeletal pain at follow-up with analysis adjusted for pain in the arms/legs at baseline**

| **Logistic regression of the association between internalizing at baseline and musculoskeletal pain onset at follow-up** | | |
| --- | --- | --- |
| **Adjusted analysis*** | | |
| **Musculoskeletal pain at follow-up** | **Odds ratio** | **95% CI** |
| Overall (N = 3,865) | 1.22 | 0.95, 1.57 |
| *Analysis adjusted for Smoking, Marijuana use, Drug use, Physical activity and Baseline pain | | |

**Results of the analysis of the association between externalizing symptoms at baseline and musculoskeletal pain at follow-up with analysis adjusted for pain in the arms/legs at baseline**

| **Logistic regression of the association between externalizing at baseline and musculoskeletal pain onset at follow-up** | | |
| --- | --- | --- |
| **Adjusted analysis*** | | |
| **Musculoskeletal pain at follow-up** | **Odds ratio** | **95% CI** |
| Overall (N = 3,865) | 1.60 | 1.21, 2.11 |
| *Analysis adjusted for Smoking, Marijuana use, Drug use, Physical activity and Baseline pain | | |
